# 10 Questions and 4 experts on Corona: Answers by Akiko Iwasaki, Gérard Krause, Samuel Alizon, and Rino Rappuoli

**DOI:** 10.15252/emmm.202012317

**Published:** 2020-04-24

**Authors:** Celine K Carret

**Affiliations:** ^1^ EMBO Molecular Medicine Heidelberg Germany

**Keywords:** Microbiology, Virology & Host Pathogen Interaction, S&S: Ethics

## Abstract

A multi‐person interview on the unrolling corona pandemic with Samuel Alizon, Akiko Iwasaki, Gerard Krause and Rino Rappuoli.

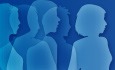

E**MM:** Following Italy, many other European countries and the USA are implementing various degrees of quarantine/social distancing to slow down the spread of the virus. Are drastic lockdown measures as applied in Huabei better than enforced social distancing to halt COVID‐19? Are these measures applied too late to have optimal impact? What would it take for other countries to escape the dramatic escalation we see in Italy or the USA?

**Gérard Krause (GK):** We are looking into a situation that will only unfold fully over the next 12–24 months. Therefore, assessment of what the better approach is also quite risky at this point.

**Samuel Alizon (SA):** To date, we are lacking detailed models to know for sure. Regarding “optimality”, there is a strong policy matter involved because one strategy can be to slow the virus spread in the population to achieve the herd immunity threshold (~ 60% for COVID‐19) through natural immunity and another is to halt its spread as long as possible for a safe and efficient vaccine to be ready to implement. Furthermore, factoring in the health cost of the lockdown itself is as crucial as it is difficult.

**Rino Rappuoli (RR):** I believe that all western countries initially underestimated the impact that COVID‐19 was going to have on the healthcare system, mortality and the economy. Quarantine and social distancing work much better if rigorously implemented very early, before the cases are widespread. At this point, all countries are implementing measures similar to those implemented in Italy.

**EMM:** Many Asian countries and regions appeared better prepared for the outbreak and to date report a lower rate of increase in cases (in particular Taiwan and Hong Kong) or decreasing numbers (Mainland China, Korea). What did they do right from the beginning?

**Akiko Iwasaki (AI):** These countries were a lot better prepared to deal with outbreaks. Hong Kong in particular has had long‐standing preparedness with epidemics. China implemented aggressive quarantine measures relatively early on. Korea quickly responded by conducting massive testing and quarantining infected people. Taiwan also responded quickly and systematically to implement rapid testing and containment. The key to dealing with epidemics is to act as early as possible because the virus spreads exponentially among an immunologically naïve population. Some key measures include hand washing, social distancing, testing widely, quarantining infected people, contact tracing and multiple retesting before lifting quarantine.

**Gérard Krause** is a medical epidemiologist. After his doctoral degree 1993 in Heidelberg, he worked in internal and tropical medicine, hospital hygiene and epidemiology in Germany and the USA. Between 2000 and 2013, he was unit head and Director at the Robert Koch Institute, Berlin. After his habilitation 2005, he became full professor at the Hanover Medical School in 2011 and department head at the Helmholtz Centre for Infection Research, Braunschweig and since 2016 also Director at TWINCORE, Hannover. Photograph: © Helmholtz Centre for Infection Research (HZI), Braunschweig

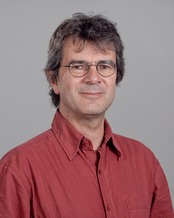

**RR:** These countries were very rigorous in implementing and enforcing from the very beginning the measures required to reduce the spread of the virus.

**EMM:** There is evidence for superspreader events at the beginning of the Korean epidemic and the European pandemic (e.g. at a bar in Ischgl, Austria). Does pandemic spread require such superspreader events? Would this have been preventable with better preparation and quick reaction such as in Taiwan?

**SA:** Heterogeneity in individual transmission is an interesting phenomenon. During the early stages of an outbreak, this heterogeneity increases the probability that the pathogen goes extinct. Indeed, for the same average basic reproduction number (R0), the presence of a superspreader also means that many hosts will cause zero secondary infections. Conversely, once the outbreak has escaped stochastic extinction, the same heterogeneity will increase the speed of spread. In summary, to prevent the emergence of an outbreak, biodiversity (e.g. of animal hosts) could offer more protection than genetic uniformity. Conversely, once the epidemic is there, decreasing superspreading events is essential.

**Akiko Iwasaki** is a Waldemar von Zedtwitz Professor of the Department of Immunobiology and Department of Molecular, Cellular, and Developmental Biology at Yale University and an investigator at the Howard Hughes Medical Institute. She earned her doctoral degree in immunology from the University of Toronto. Iwasaki did her Postdoctoral Fellow at the National Institutes of Health. Photograph: © Yale University

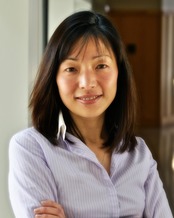

**RR:** Although I am aware of anecdotal reports, I am not aware of documented cases of superspreaders. In my mind, we do not need superspreaders to explain the propagation of this pandemic. Probably, the silent spread of asymptomatic patients is more important.

**EMM:** How far are we from a vaccine and what are currently the most promising vaccine approaches to SARS‐CoV‐2 in terms of availability and efficacy?

**AI:** We will not know whether a vaccine is effective for a year or so. The phase 1 trial for the mRNA vaccine has already begun in the USA. Since no vaccines have been tested even in animal models for efficacy, it is difficult to know what the most promising approaches are yet. The problem is that we really don't know the ideal immune response that would confer best protective response against SARS‐CoV‐2 and how best to achieve it.

**GK:** I am not an immunologist or virologist, but my experience from similar events in the past is that at the beginning of an epidemic, we tend to have too optimistic estimates about how quickly a vaccine could be available. For Coronavirus, this may even be more difficult.

**RR:** Under normal conditions, vaccine development requires 15–20 years. This is mostly because we want to be absolutely sure that vaccines are safe and effective. When there is an emergency, the risk/benefit ratio changes and vaccine development can be accelerated. In the case of Ebola, 5 years were sufficient to develop and licence a vaccine. For COVID‐19, the new technologies and the experience gained with Ebola are going to help to further accelerate the development of a vaccine; however, I believe it will be difficult to get any available to people before at least 12 months.

The following are the vaccines that are being developed:

• RNA vaccines: These can be developed very quickly (in 2013, we had a vaccine against H7N9 to immunise mice in 1 week). The platform is not yet mature, there is no vaccine approved with this technology, and safety is still in question. However, depending on the risk/benefit ratio, it could be the fastest approach. Indeed, an RNA vaccine against SARS‐CoV‐2 is the first one to go to phase I clinical trial.
• Viral vectors (Adeno, ChAd, measles, VSV….): The platform is more mature than RNA, and it is easy to splice a synthetic gene into a vector. One product (Ebola) is already licensed, others are in phase III, and safety is reasonable. Some manufacturing capacity is available. This can also be fast.
• Traditional protein‐based vaccines: the recombinant spike protein to be expressed in mammalian cells, baculovirus or plant cells +/− adjuvants. The spike protein will probably be engineered to stabilise the prefusion form. I am sure this technology is going to work. Initially, it will require more time to get to phase I, but given the experience we have in developing and manufacturing protein‐based vaccines, this is probably the technology we need to count on, if we need a very large number of vaccine doses. Very likely, any protein‐based vaccine will require an adjuvant to improve the immunogenicity and for dose sparing.

Finally, I believe we should develop Human monoclonal antibodies starting from PBMCs from convalescent people. These can be extremely important for therapy and prevention. In the case of Ebola, they were the fastest therapeutic tool to be developed.

**EMM:** What is known about the population immune response to SARS‐CoV‐2? Some have argued that exposure of the general population is needed to build up herd immunity. Is this valid for COVID‐19?

**AI:** It is too early to know whether naturally acquired immune response to COVID‐19 is sufficient to prevent future infection with the same virus and for how long. Relying on natural infection to build up herd immunity is very dangerous, and the risk of even “low‐risk” individuals becoming critically ill and dying is too high to implement such a strategy.

**SA:** Immunity to SARS‐CoV‐2 is still poorly known. It appears to be effective so far because there are not really cases of reinfection documented. The problem is to know how long the immunity will last. Herd immunity is the only way to avoid a future epidemic. It can either be built through natural immunisation or through vaccination if a vaccine is developed. Importantly, even with the goal of achieving herd immunity via natural immunisation, it is essential to control the epidemic: first, to protect individuals who are most at risk; second, to allow hospitals to cope with the epidemic waves. Third, because even if the herd immunity threshold is ~ 60% for COVID‐19, if the epidemic grows unchecked, 90% of the population are likely to become infected (both these numbers depend on R0 which varies across regions).

**EMM:** What is the chance of a second infection wave in Wuhan, in the rest of China or elsewhere once the first peak has passed? Recent data published in non‐primate rhesus monkeys suggested at least 3 months lasting immunity. Do we have any human indication of acquired immunity?

**SA:** If all control measures are lifted before the herd immunity threshold is reached, a second wave is likely. However, maintaining looser control measures could decrease the R0 sufficiently to slow down a future epidemic spread and make it more controllable by targeted policies. The quasi‐absence of documented reinfection points to the existence of acquired immunity. The question is, how long does it last? Earlier data on SARS‐CoV‐1 suggest it might not be as long‐lived as we would hope.

**Samuel Alizon** is a CNRS Research Director based in the MIVEGEC department in Montpellier, France. He is an evolutionary ecologist specialised in the modelling of infectious disease dynamics. Alizon earned his PhD in 2006 at Université Paris 6 and his habilitation in 2013 at Université Montpellier 2. Photograph: © Samuel Alizon

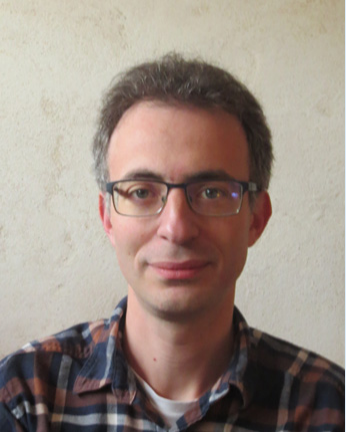

**RR:** We know very little about acquired immunity to COVID‐19. Studies in macaques have shown that reinfection does not occur in previously infected animals, possibly suggesting that herd immunity can be built in principle.

**EMM:** What is different in children's and younger adults’ immune system in whom the infection is largely asymptomatic? Is this an argument for wider testing?

**SA:** Clearly, it would be interesting to know more about the role of children in spreading the infection to adjust national policies, such as school closures.

**RR:** We know that the infection is very mild in children and young adults, and that the severity of the disease increases with age, becoming life‐threatening above 80 years of age. The severe disease is mostly caused by a cytokine storm produced by immune cells that migrate to the lungs to combat the virus. Children usually have a lower cellular response, and this could explain the milder disease. Wide testing makes sense to follow the contacts of cases or in small populations. When an entire country is affected, wide testing becomes hard to implement.

**EMM:** As some reports suggest that the R0 and fatality rate of SARS‐CoV‐2 is likely to be higher than Spanish flu, why do we currently see a much lower death rate?

**SA:** There is always a social component to an epidemic and perhaps even more to a pandemic. In the case of the 1918–1919 influenza pandemic, the world was exiting a World War and the average health in the populations was much lower than it is today. Furthermore, it is possible that a significant fraction of the mortality was due to coinfection by bacterial infections (antibiotics were not available then).

**RR:** To my knowledge, the R0 of the 1918 Spanish Flu was not lower than the one of COVID‐19 which has been estimated to be 2.6. We do not know the reason for the high mortality of the Spanish Flu, but secondary bacterial infections and lack of hygiene may have increased the lethality of the infection.

**EMM:** Why do we see a low fatality rate in Singapore, Hong Kong, Japan and South Korea despite high infection levels compared to higher death rates in Italy, Spain or the USA?

**GK:** Those are different stages in different countries and many other differences that we are looking into currently. Age distribution seems to play a relevant role.

**SA:** One should always bear in mind that fatality rate depends on the total number of cases tested. Therefore, the more a country screens for COVID‐19, the lower the case fatality rate will be.

**Rino Rappuoli** is Chief Scientist and Head of External Research and Development (R&D) at GlaxoSmithKline (GSK) Vaccines. Previously, he has served as visiting scientist at Rockefeller University and Harvard Medical School and held roles at Sclavo, Vaccine Research and CSO, Chiron Corporation, and Novartis Vaccines. Photograph: Wikipedia/Public Domain

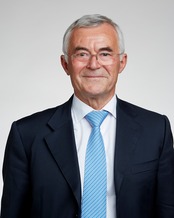

**RR:** It is too early to be able to understand the cause of the different death rates. Different testing procedures can explain some of the inconsistencies; however, different age of the population can also contribute.

**EMM:** Is COVID‐19 mutating faster than other similar viruses? Could this be related to different lethality/infectivity rates?

**RR:** COVID mutates, but for the moment it is not particularly fast.

**EMM:** Is it currently possible to estimate how long the pandemic will last in a given country at this time? What data are needed for a definitive prediction?

**GK:** Such predictions are very difficult.

**SA:** We could estimate the duration in the absence of any public health policy but, fortunately, governments are reacting to mitigate this spread. At best, we could predict the effect of these policies. On a more worrying trend, some actions in response to these policies (e.g. people fleeing large cities as soon as travel restrictions are announced) could have the adverse effect that are even more difficult to anticipate.

**RR:** The only information we have is that in China, Korea and Singapore, we have been able to contain the first wave of COVID‐19. However, the majority of the population in these countries is still susceptible to infection. Given that this virus is already present in most countries worldwide, it is unlikely that it will disappear. We may see secondary waves of infections, and eventually, it may become endemic. My hope is that by that time we will be ready with vaccines and drugs.

